# *Acinetobacter*: an emerging pathogen with a versatile secretome

**DOI:** 10.1038/s41426-018-0030-4

**Published:** 2018-03-21

**Authors:** Noha M. Elhosseiny, Ahmed S. Attia

**Affiliations:** Department of Microbiology and Immunology, Faculty of Pharmacy, Cairo University, Cairo, 11562 Egypt

## Abstract

*Acinetobacter baumannii* is a notorious pathogen that has emerged as a healthcare nightmare in recent years because it causes serious infections that are associated with high morbidity and mortality rates. Due to its exceptional ability to acquire resistance to almost all available antibiotics, *A. baumannii* is currently ranked as the first pathogen on the World Health Organization’s priority list for the development of new antibiotics. The versatile range of effectors secreted by *A. baumannii* represents a large proportion of the virulence arsenal identified in this bacterium to date. Thus, these factors, together with the secretory machinery responsible for their extrusion into the extracellular milieu, are key targets for novel therapeutics that are greatly needed to combat this deadly pathogen. In this review, we provide a comprehensive, up-to-date overview of the organization and regulatory aspects of the *Acinetobacter* secretion systems, with a special emphasis on their versatile substrates that could be targeted to fight the deadly infections caused by this elusive pathogen.

## Introduction

Medically relevant members of the genus *Acinetobacter* are increasingly recognized as a serious health hazard worldwide^1^. In particular, *Acinetobacter baumannii*, which is a member of the ESKAPE group of pathogens (*Enterococcus faecium*, *Staphylococcus aureus*, *Klebsiella pneumoniae*, *Acinetobacter baumannii*, *Pseudomonas aeruginosa*, and *Enterobacter* species), is a leading cause of nosocomial infections^2^. The group showing the highest prevalence of these infections includes immunocompromised and debilitated patients confined to intensive care units. As an opportunistic pathogen, *Acinetobacter* takes advantage of the weakened immune system of this patient group, attacking different body tissues and causing a variety of infections. The most relevant *Acinetobacter* infections include ventilator-associated pneumonia (VAP), catheter-related bacteremia, wound and soft tissue infections (particularly in burn wards), urinary tract infections, post-surgical endocarditis, and meningitis^3^. *Acinetobacter* infections are not restricted to hospital settings, with community-acquired infections being increasingly reported^3^. Risk factors include geographical locations (tropical, sub-tropical, and humid climates) and underlying health conditions, such as diabetes mellitus, chronic obstructive pulmonary disease, excessive smoking, and alcohol consumption^4^.

The challenge in treating *Acinetobacter* infections is primarily attributed to its high intrinsic tolerance to most antibiotics. An exceptional low permeability to antibiotics, constitutively expressed efflux pumps, resistance genes harbored on genetic islands, and high genetic plasticity are all mechanisms employed by *Acinetobacter* to combat antibiotics^5,6^. In a recent report, approximately 37% of all assayed *A. baumannii*–*calcoaceticus* complex isolates were resistant to imipenem and meropenem, the drugs of choice for VAP^7^. Polymyxins have remained effective against *Acinetobacter*, but because therapeutic doses of these antibiotics are nephrotoxic and neurotoxic, they remain a last treatment option^3^. An alternative approach of repurposing drugs already in the market, such as the anticancer drug mitomycin C, is currently under study^8^. Another approach is employing novel combinations of drugs, such as daptomycin-colistin-teicoplanin, intensified meropenem-polymyxin B, or using novel analogs of conventional antibiotics, such as tetracyclines^3,9,10^. However, despite continued trials to maximize the use of available antibiotics and drugs, their toxicities are not eliminated. Moreover, the efficacy of such combinations have not been fully validated. Discovering novel, druggable targets requires a more profound understanding of the resistance, and virulence mechanisms of *Acinetobacter* species.

Anti-virulence drugs could be a promising alternative to conventional antibiotic therapies. By targeting the virulence factors of a pathogen rather than their ability to growth, less pressure is exerted on microbes to develop resistance^11^. Recently, trials have begun to develop anti-virulence drugs with targets that include proteases, bacterial adhesins, and transcription factors, such as LysR^11–13^. As for *Acinetobacter*, despite the knowledge that has been compiled over the past decade about its pathogenesis, still little is known about its virulence attributes and how they are employed to cause disease. In this regard, secreted proteins are intriguing therapeutic targets because of their crucial role in microbial proliferation within infected tissues and for manipulating host defenses^14^. Gram-negative bacteria use complex membrane-bound structures, termed secretion systems, for the specialized secretion of biologically active proteins. These systems can span both the inner and outer membrane (IM and OM, respectively), such as the type I secretion system (T1SS), T2SS, T3SS, T4SS, and T6SS, or span the OM only, as observed for the T5SS. Whether the secreted protein is ejected into the extracellular space, remains cell-bound, or becomes injected into the target host cell depends on which secretion system is used^15^. Because of their importance in the secretion of microbial virulence factors, survival, fitness, and competition, secretion systems and their substrates represent excellent therapeutic targets. For these reasons, increased attention has been recently paid to the roles of these systems in the pathogenicity of *Acinetobacter*. In this review, the roles of various secretion systems and their substrates in the virulence of this pathogen are discussed.

## Type VI secretion system

T6SSs aid various Gram-negative pathogens in establishing a favorable niche by eliminating competing bacterial species. The T6SS functions as a “molecular syringe” that injects a number of toxins into the target competitor cell, interfering with vital cellular processes to achieve bacterial killing^16^. Species harboring a T6SS are able to secrete two major proteins, hemolysin co-regulated protein (Hcp) and valine glycine repeat protein (VgrG), which become membrane-associated to form the tail tube^17^ and capping spike, respectively, and are used to deliver, pierce, and inject effector proteins^18^. The potent toxins delivered by T6SSs can be used to attack either prokaryotic or eukaryotic cells (sometimes both), with targets that include bacterial peptidoglycans, nucleic acids, and cell membranes through lipase/phospholipase and pore-forming activities^19^. Secreting cells are protected from the actions of the antibacterial effectors through specific immunity proteins. These proteins are always encoded adjacent to the effectors, and following expression, reside in the cellular compartment in which the toxin exerts its effect^19^.

The T6SS is by far the most comprehensively studied secretion system of *Acinetobacter*. The genes encoding the core components of the T6SS were first identified through a homology search in a study investigating how *A. baumannii* compensates for the lack of membrane lipopolysaccharide (LPS) in an isogenic *lpxA* mutant^20^. Eleven of the 106 genes downregulated in the *A. baumannii* ATCC 19606 LPS mutant were consecutive, and located adjacent to one another on the chromosome (HMPREF0010_01111 to HMPREF0010_ 01125). Through bioinformatic analyses, these genes were identified as part of a putative T6SS-encoding locus consisting of 17 genes. Twelve of these genes were predicted to encode proteins homologous to T6SS components in other bacterial species (*tssABCDEFHJLMNO*), whereas five proteins were unique to *A. baumannii* (*tssGIKPQ*). The study was also the first to show the functionality of the T6SS in *A. baumannii*. The authors could identify the Hcp protein in the culture supernatants of the wild-type strain, whereas it was absent from the supernatants of the LPS-deficient mutant, in agreement with findings from the transcriptomic data^20^.

These initial insights were followed by studies from two research groups to genetically and functionally characterize the system. In the first work, by Weber et al.^21^, the authors showed that the T6SS was functional in a number of *A. baumannii* strains and *Acinetobacter* species by assessing the production of Hcp. Interestingly, secreted Hcp levels varied between the strains, with the highest observed in strains SDF, 19606, and 1224, and the lowest in AYE and 1375, which showed almost no detectable secreted Hcp. By constructing Δ*hcp* and Δ*tssM* knockout mutants, the authors showed that the secretion of Hcp occurs in a T6-dependent manner. Carruthers and co-workers^22^ used similar approaches to characterize the functionality of a T6SS in *A. nosocomialis* strain M2. However, both studies reached contradictory conclusions with respect to the role of T6SS in the virulence of *A. baumannii*. In the first study by Weber et al.^21^, three different infection models (*Dictyostelium discoideum* amoebae, *Galleria mellonella* waxworms, and a murine pneumonia model) showed no significant difference between the ability of both the wild-type and Δ*tssM* mutant to cause infection. In addition, the system did not appear to provide *A. baumannii* with an antibacterial advantage in a *Escherichia coli* MG1655 killing assay. Studies using different growth conditions or types of prey still presented no evidence of the antibacterial role of T6SS in *A. baumannii*. The authors concluded that *A. baumannii* may require specific conditions for the T6SS to be active, be highly specific for attacking certain prey, or that it had other functions^21^. Conversely, Carruthers and co-workers^22^ showed that *A. nosocomialis* M2 could kill *E. coli* DH10B in killing assays in a T6SS-dependent manner. Since the killing ability of *A. baumannii* was diminished when separating it from the *E. coli* prey by a membrane filter, the authors concluded that the killing was contact-dependent^22^. Repizo and co-workers^23^ showed that *A. baumannii* strains ATCC 17978 and DSM30011 could both kill *E. coli*. However, inactivating the T6SS abolished this ability only in DSM30011 and not ATCC 17978. They also observed that *A. baumannii* DSM30011 could outcompete other nosocomial pathogens that infect the respiratory tract, including strains of *Pseudomonas aeruginosa* and *Klebsiella pneumoniae*, even when these strains possessed an active T6SS of their own^23^. *A. baumannii* T6SS+clinical isolates showing detectable levels of Hcp secretion were more frequently detected in infected immune-suppressed patients, recipients of immunotherapy, transplants, or patients connected to IV-lines and catheters. As expected, these isolates formed better biofilms and were more resistant to human serum killing than the T6SS-containing isolates^24^.

In an exquisite study by Shneider et al.^18^, it was shown that proline-alanine-alanine-arginine (PAAR) proteins bind to the VgrG proteins at the end of the T6SS tubular organelle. These proteins, whose encoding genes are typically observed downstream from *vgrG*-like genes, fold into cone-shaped structures that sharpen the tip of the T6SS spike. Inactivation of all the genes encoding PAAR-like proteins in *Acinetobacter baylyi* ADP1 reduced the secretion of Hcp by a remarkable 90%. Interestingly, inactivation of these genes also led to a 10,000-fold reduction in the ability of *Acinetobacter* to kill *E. coli* MG1655, in agreement with the proposed role of T6SS in bacterial competition in the previous study. Single mutants in the PAAR encoding genes did not show an equivalent defect in T6SS function in *Acinetobacter* ADP1, indicating that the loss of any one PARR protein could be compensated for by the remaining proteins. A plasmid-expressed vesicular stomatitis virus G glycoprotein epitope-tagged PAAR protein (ACIAD2681) was successfully secreted by *Acinetobacter* ADP1 and restored the *E. coli*-killing ability of the PAAR protein triple mutant as well. This indicates that fusing other proteins with a PAAR motif is likely to recruit them for T6SS-dependent secretion. Of the seven subclasses of PAAR proteins identified by Shneider and co-workers^25^ in the previous study, *A. baumannii* was observed to harbor members from classes 1, 2, and 5 in its genome. A remarkable variation in the number of PAAR proteins in each *A. baumannii* strain was observed, with strain D1279779 having none (exhibiting a lack of T6SS functionality) and strain SDF possessing 10 PAAR protein homologs^25^.

How the T6SS is regulated in *A. baumannii* is not fully understood; however, some regulatory elements have been elucidated. A hypermotile variant of strain ATCC 17978 was observed to have an insertional disruption in a gene encoding a histone-like nucleoid structuring protein (H-NS), which is a known transcriptional regulator^26^. This mutation lead to a considerable upregulation of the T6SS gene cluster (A1S_1292-1311), approximately 50-fold higher than the wild-type strain. It is worth mentioning that the hypermotile strain was also observed to demonstrate increased killing of *Caenorhabditis elegans* nematodes, elevated pellicle formation, and adherence to the eukaryotic A549 pneumocytes. However, VgrG and PAAR proteins were shown to be not under the regulation of H-NS in this study^25^. The downregulation of the T6SS cluster in an LPS-deficient *A. baumannii* strain was likely attributable to the lack of this membrane-stabilizing element from the OM, or possibly to stabilize the defective OM by reducing membrane stress^20^. Light was observed to induce the expression of the T6SS gene cluster in ATCC 19606, along with a number of other virulence determinants^27^. Why the virulence and physiology of *A. baumannii* would be light-dependent is not totally understood, yet the effect was observed to be strain-dependent. A key study by Weber et al.^28^ then revealed a unique regulatory aspect of the T6SS in *A. baumannii*, which perhaps accounted for the discrepancy noted earlier in the role of this system in virulence. Two variants of the same *A. baumannii* strain isolated from a patient with a polymicrobial infection had two contrasting Hcp secretion patterns. The Hcp-secreting variant, considered T6SS+, could kill *E. coli* and *K. pneumoniae* isolated from the polymicrobial infection as well as other laboratory *E. coli* strains. Meanwhile, the non-Hcp-secreting variant was not able to compete with them in the killing assays. Whole-genome sequencing revealed that the T6SS+ variant was lacking 170 kb of DNA when compared to the T6SS− variant. The T6SS+ variant was also highly sensitive to antibiotics, which is logical given that the missing piece of DNA was predicted to carry a number of antibiotic resistance determinants. Furthermore, the authors showed that the T6SS+ *A. baumannii* strain did not target its T6SS− variant, yet it could kill non-kin *A. baumannii* T6SS− cells. This led to the hypothesis that *A. baumannii* could express immunity proteins that protect it from T6SS-mediated killing, which have yet to be identified. The authors then revealed that the additional DNA element in T6SS− variants was in fact a plasmid (pAB04-1 in AB04 and pAB3 in AB17978), which is self-transmissible. These plasmids carried two transcriptional regulators, *tetR*-like1 and *tetR*-like2, which could efficiently suppress Hcp expression in T6SS+ strains when ectopically expressed. It is worth mentioning that an *hns*-like gene was also observed in each plasmid, although its ectopic expression did not affect Hcp production.

A RNA-sequencing transcriptomic analysis on *A. baumannii* cells with or without the repressing plasmid identified novel genetic elements needed for a functional T6SS^29^. Mutants in the three open reading frames in *A. baylyi* ADP1 (ACIAD2685, ACIAD2693, and ACIAD2699) showed detectably lower levels of Hcp in cell pellets, and total absence from culture supernatants using western blots. Interestingly, despite being conserved across the genus, the identified genes have no homologs in other T6SS harboring species, which might indicate that they are unique to the T6SS in *Acinetobacter*. The authors used bioinformatics to predict T6SS effectors located in proximity to each of the four VgrG encoding genes in ATCC 17978, as well as their potential immunity factors. Apart from *vgrG1* and its associated effector *tle*, other *vgrG* genes and their associated effectors showed modest or no significant transcriptional differences between T6SS+ and T6SS− strains. Mutants lacking VgrG1, solely or in combination with other VgrG proteins, had significant defects in Hcp secretion, whereas a loss of the other three VgrG proteins (VgrG2, VgrG3, and VgrG4) maintained wild-type Hcp levels. These findings showed the crucial role that VgrG1 plays in Hcp secretion in *A. baumannii*. However, it seems that maintaining wild-type levels of Hcp secretion is not always directly correlated to the antibacterial activity of *A. baumannii*. While the loss of both VgrG2/VgrG4 and VgrG3/VgrG4 maintained Hcp levels and antibacterial activity, the loss of all three VgrG proteins maintained Hcp levels but led to a loss of antibacterial activity. These results indicated that secretion of the effectors Tde and Tse, proximal to *vgrG2* and *vgrG3*, respectively, is essential for the killing process. The authors confirmed this by testing the antibacterial activity of the Δ*vgrG34* Δ*tde* and Δ*vgrG24* Δ*tse* mutants, which was observed to be abrogated in both. From the novel elements identified by the transcriptional and proteomic analysis to correlate to a functional T6SS, the protein designated as TagX (type VI-associated gene X) is particularly interesting. Bioinformatic analysis of this protein revealed that it has a C-terminal peptidase domain resembling that of the PG-cleaving peptidase VanY superfamily of proteins (pfam13539). In addition, it shares the active site and metal coordination residues with Ply500, which is an l-alanoyl-d-glutamate endopeptidase previously studied in *Listeria*^30^. This suggested that TagX may have a PG-degrading activity, which could be used to allow the assembled T6SS to be mobilized across the PG layer of the cell wall. Purified TagX, but not its active site mutant protein TagX^D287N^, could degrade PG. The activity was higher using the purified C-terminal fragment of the protein and the C-terminal-containing degradation products than the full-length TagX. This showed that only the C-terminal part of the protein has the enzymatic domain necessary for the PG-cleaving activity. Why the full-length TagX does not degrade PG as efficiently as the C-terminal fragment is not fully understood, yet the authors speculated that the N-terminal domain might have some auto-inhibitory function. The authors also speculated that interaction with other T6SS components might regulate the activity of TagX. Investigating the cleavage mechanism by monitoring the degradation products of the TagX reaction with PG by high-performance liquid chromatography and mass spectrometry (MS) yielded the PG fragment *N*-acetylglucosamine-*N*-acetylmuramic acid attached to l-Ala. This suggests that TagX cleaves the bond between the l-Ala and d-Glu of the PG pentapeptide, acting as an l, d-endopeptidase.

The crystal structure of the Hcp of *A. baumannii* was solved to a 1.55 Å resolution to understand more about the function of the T6SS in this microbe^31^. The study showed that despite the low sequence similarity between the Hcp from *A. baumannii* and those of other species, the structure is almost identical. The overall structure consists of two β-sheets forming a tight β-barrel, which are flanked by an α-helix and an extended loop. The only significant difference is in the length of the loops connecting the strands, and in this regard, Hcp1 of *P. aeruginosa* is the closest match. This similarity suggests that both proteins will be closely related in their secretory functions. However, in contrast to previously characterized members, the crystal packing of the Hcp of *A. baumannii* is quite unique, with the nanotube rings interacting both head-to-head and head-to-tail. The implications of this unique organization are not yet understood.

## Type II secretion system

The T2SS was the first secretion system to be characterized^32^, and over the years it has been extensively studied in various bacterial species. Twelve genes, often designated *gsp* for “general secretory pathway” and denoted C, D, E, F, G, H, I, J, K, L, M, and O, encode the core components of the T2SS^33^. These components form a complex apparatus that spans both the IM and OM^15,34^. A pentadecamer of the T2SS protein “D” forms the “secretin,” which is the OM component that forms the “pore” through which proteins are secreted. The T2SS proteins C, F, L, and M form the IM scaffold, with the T2SS C protein acting as the connection between this IM complex and the OM secretin. With this structure as the base, a pseudopilus is formed consisting of polymerized major pseudopilin protein G and capped with minor pseudopilin proteins H, I, J, and K, spanning the periplasm. An ATPase consisting of a T2SS E protein hexamer is connected to the IM platform on the cytoplasmic side. The last core component is the “O” protein, which acts as an IM prepilin peptidase and cleaves and methylates the major pseudopilin protein G before being recruited to the pseudopilus. An accessory component that is present in some species is a chaperone protein called the “pilotin,” which is responsible for the assembly of the secretin and its targeting to the OM. Various genes have been shown to encode this protein, such as *gspS*, *aspS*, and *yghG* depending on the species^35^. The T2SS components A, B, and N are reported to play a role in peptidoglycan binding to aid in the multimerization of the secretin in some species^36^.

Secretion of proteins through the T2SS is a two-step process that first involves the passage of the protein through the IM to the periplasm using the sec translocon or the twin arginine translocation (tat) pathway. After folding into the tertiary structure, the protein is translocated across the OM through the T2 apparatus^15^.

Extensive study has shown that the T2SS is dedicated to the secretion of a large number of proteins, contributing to the virulence of many human pathogens, including members of the *Acinetobacter*, *Aeromonas*, *Escherichia*, *Klebsiella*, *Legionella*, *Pseudomonas*, *Stenotrophomonas*, and *Vibrio* genera^37^. Parche et al.^38^ were the first to describe the role of “XcpR” as a T2SS component in the degradation of dodecane in *A. calcoaceticus*. Johnson et al.^39^ identified the components of a T2SS in *A. baumannii* ATCC 17978 through a homology search based on the T2SSs in *Vibrio* and *Pseudomonas* species. The genetic arrangement of the system in *A. baumannii* is unique, with the genes arranged in divergent clusters scattered across the chromosome rather than in a single operon, as is observed in other species. The group showed that the system was functional and was required for the secretion of substrates that could degrade lipids, since mutants in *gspD* and *gspE* were unable to grow in minimal media supplemented with olive oil as a sole carbon source. Mutants in other system components, including *gspN* and another probable ATPase, *gspE2*, did not show this growth defect, suggesting that they are dispensable for the system. Another explanation for this redundancy in the genes encoding T2SS components is the probable overlap between T2SS and T4 pilus system-encoding genetic loci on the chromosome of *A. baumannii*. The chromosome of *A. baumannii* harbors genes similar to those encoding LipA and LipB, known type II substrates in *Vibrio* cholerae^40^. A mutant in *lipA* could not grow on olive oil, and complementing the T2SS mutant with a plasmid expressing LipAB did not reverse this defect, suggesting that their secretion is T2SS-dependent. By measuring the activity in culture supernatants and through immunoblotting assays, the authors also showed that these lipases were not expressed unless a lipid substrate was present in the medium. LipA is dependent on its chaperone, LipB, since Δ*lipA* strain complemented with a LipAB-encoding plasmid showed a higher extracellular lipase activity than with LipA complementation alone. Targeting LipA during its expression with the serine hydrolase probe ActiveX also showed that it is a serine hydrolase belonging to the group I lipases. Orlistat, an obesity drug that inhibits the activity of human pancreatic lipase, was also able to inhibit the activity of LipA. Both Δ*gspD* and Δ*lipA* showed a defect in colonization relative to the wild-type strain in a systemic infection model.

The genetic arrangement of the T2 components in *A. nosocomialis* followed the same cluster pattern as in *A. baumannii*, with slight differences in the order of genes^41^. The authors used sodium dodecyl sulfate-polyacrylamide gel electrophoresis to compare the wild-type and Δ*gspD* strains to demonstrate that the system contributes to the pool of secreted proteins of *A. nosocomialis*. Furthermore, two-dimensional gel electrophoresis analysis was used to better resolve the protein bands and assay the T2SS secretome. The top secreted candidates included CpaA metallopeptidase, LipA, and an additional α/β-hydrolase not previously characterized. Since the chromosomes of *A. baumannii* and *A. nosocomialis* bear a single gene that could encode a prepilin peptidase, the authors questioned whether it could commonly function with both the T2SS and T4 pili system, as both are evolutionarily related^33^. The prepilin peptidase PilD could process the major pseudopilin protein GspG, resulting in its higher electrophoretic mobility. The authors confirmed the T2SS-dependent secretion of CpaA by detecting the His-tagged version of the trans-expressed protein only in the secreted fractions of strains having a functional T2SS. They also characterized the gene downstream of the *cpaA* open reading frame (ORF) as encoding a chaperone necessary for its secretion, designating it CpaB. LipA was identified as part of the T2SS secretome of *A. nosocomialis* and is homologous to the LipA characterized in ATCC 17978 and is similarly dependent on the chaperone LipB for its activity. It is important to stress that the Δ*gspD* mutant almost totally lacked any lipase activity. This observation indicates that the lipase activity in the supernatants is almost completely dependent on T2SS, highlighting the importance of this system for lipase secretion in *Acinetobacter*. A bioinformatic analysis showed that many T2SS substrates are dependent on membrane-bound chaperones encoded closely upstream or downstream of the substrate gene, as is the case for LipB and CpaB. It is interesting to anticipate whether this chaperone family could show common features that could be used to identify the diverse T2SS substrates, which often vary in sequence and structure. The study identified an additional T2SS substrate, LipH, as a novel protein with lipase activity that is also dependent on the T2SS for secretion. As with ATCC 17978, the T2SS was needed for optimal virulence and colonization by *A. nosocomialis* in a *G. mellonella* survival model and a murine pulmonary infection model, respectively.

A third study investigated the nature of the T2SS-encoding loci in *A. baumannii*^42^. The predicted genetic arrangement differed slightly from that previously published, and two genes were annotated as *gspG*. One of these genes is predicted to encode a protein that bears the conserved prepilin peptidase-processing signal (Gly*Phe Thr Leu). Interestingly, Johnson et al.^39^ annotated this protein as a GspH in their study. In addition, in the work by Weber and coworkers Weber et al.^41^, the protein they annotated as GspH was originally annotated as a member of the TetR family of transcriptional regulators in the NCBI database. This raises a question as to whether the T2SS in *Acinetobacter* has a GspH component, and if this protein was properly annotated in the aforementioned studies. The study also classified the system in *A. baumannii* as non-pilotin-dependent (not requiring the GspS chaperone). A phylogenetic tree based on the amino acid sequence of GspD placed it with the non-pilotin-dependent group, and the Xcp T2SS system of *P. aeruginosa* was the closest relative. The genome of *A. baumannii* also lacked genes that encode proteins homologous to the known families of pilotins. Since no experimental evidence was provided, the authors hypothesized that *Acinetobacter* could rely on a new family of chaperones that has a unique amino acid sequence, which has yet to be identified. MS was used to identify the most prominent band absent from the secreted fractions of the Δ*gspD* mutant, a lipase designated as LipAN. This protein, which was annotated in the proteome as a hypothetical protein, did not have a homolog in any other *A. baumannii* strain. However, it was homologous to some well-identified *C. albicans* lipases, as well as the cold-active lipases of *Psychrobacter* and *Moraxella* species. In agreement with the previous studies, the Δ*gspD* mutant showed a significant colonization defect in a pneumonia model. The wild-type strain over-expressing LipAN showed better lung colonization than the mutant or wild-type strains. This study revealed that this protein has broad substrate specificity and that it has activity against phospholipids. Finally, a T2SS secretome analysis in *A. baumannii* ATCC 17978 showed that it potentially secretes more than 40 substrates. Six of these proteins, including LipAN, are annotated as enzymes with lipid-degrading activity, demonstrating its importance for the physiology and virulence of *A. baumannii*^42^. The overall importance of the T2SS for the pathogenic process of *A. baumannii* was supported by its ability to protect it from human serum complement^43^. A mutant in the *gspD* gene was a 100-fold more sensitive than the wild-type strain in a serum killing assay. This impressive effect suggests that the T2SS could be a plausible target for drug development, and indeed, a synthetic compound library was screened for potential inhibitors of T2SS activity. Although no T2SS inhibitors were identified in this preliminary screening, the assay proved to have a great potential to be utilized in future screens with larger libraries^43^.

Thus far, the regulatory elements of T2SS expression in *A. baumannii* are unknown. The available evidence indicates that the T2SS is active under normal laboratory conditions at both the mid-log and stationary phases of growth^39,41,42^. Interestingly, the loss of LPS through a *lpxC* gene mutation not only led to colistin resistance but also affected the global transcriptomic profile of *A. baumannii* and downregulated the T2SS ATPase, as observed with the T6SS^44^. It is probable that this downregulation occurs in response to the loss of membrane integrity as a result of LPS loss, since the T2SS is an integral part of the cell membrane. An important adaptive mechanism for bacteria involves transcriptional remodeling through genetic changes that the organism undergoes during the infection and treatment processes. A recent study reported that one of these mutations observed in clinical *A. baumannii* isolates during infection was in a region harboring a gene encoding a DNA-binding protein upstream of a T2SS-encoding cluster^45^. Although none of the T2SS components showed varied expression levels as a result, many of the predicted T2SS substrates from the secretome studies were differentially expressed. Moreover, the expression of LipH, one of the T2SS substrates in *A. nosocomialis*, was elevated in the presence of human serum^41^. Although the implications of these findings cannot be fully understood at the moment, they still cast a light on the importance of gaining a better understanding of how *A. baumannii* could utilize the elements and substrates of the T2SS inside the host.

## Type I secretion system

A T1SS is defined by three components, an ATP-binding cassette membrane transporter, a membrane fusion protein present in the IM, and an OM component that varies among T1SSs^46^. The T1SS that is best characterized is the TolC-HlyD-HlyB system of *E. coli*, dedicated to the secretion of the 110 kDa HlyA hemolysin^47^. Substrates of the T1SS possess an uncleavable C-terminal secretion signal, and the secretion process takes place in one step across both lipid membranes. T1SS encoding clusters typically consist of the substrate gene, the exporter proteins, and sometimes a helper gene that promotes the function of the substrate. In case of the *hly* operon, the components include the *hlyA* hemolysin, *hlyC*, an acyltransferase that promotes the function of HlyA through lipidation, and *hlyD* and *hlyB*, which are the export proteins^48^. The OM component is typically encoded by a gene outside the operon, as is case for TolC of the Hly system. A wide range of T1SS substrates have been described and are linked to disease processes, such as the hemolysins and CyaA proteins of *Bordetella pertussis*^49^, the multitoxin MARTX Vc from *V. cholerae*^50^, and the giant adhesins LapA^51^ and SiiE from *P. fluorescens* and *Salmonella enterica*^52^, respectively.

In *A. nosocomialis* M2, a T1SS that is homologous to the TolC-HlyD-HlyB system of *E. coli* was identified using bioinformatics^53^. The altered protein secretion profile of the *tolC-hlyBhlyD*::*kan* mutant that was restored to normal upon complementation indicated that the system is functional. The authors next characterized the T1SS secretome in minimal media using cells grown to mid-log phase. The top hit was a protein encoded by ORF M215_09430, characterized by having a C-terminal secretion signal, multiple peptidase M10 Serralysin C- terminal domains, and a Ca^2+^-binding protein-RTX toxin-related domain, named RTX protein. The second candidate was a protein encoded by ORF M215_02910 and was strongly related to the biofilm-associated protein (Bap), but it lacked the C-terminal secretion signal, which could be due to a premature truncation, which is common in *bap* repetitive genes, or a faulty protein alignment from the secretome study. Other proteins lacking the C-terminal secretion domain were also differentially secreted between the wild-type strain and the T1SS mutant, including the T6SS proteins Hcp and several VgrG proteins, as well as T2SS substrates, such as CpaA, LipH, rhombosortase, and rhombotarget A. The absence of a functional T1SS only minimized Hcp secretion in minimal but not rich media. The cross-talk seems specific between T1SS and T6SS, as mutants in the T2SS and T4P prepilin peptidase *pilD* did not alter Hcp secretion. Because one of the most differentially secreted proteins by T1SS is a Bap homolog, the authors tested the role of this system in biofilm formation. Indeed, the T1SS mutant showed an attenuated biofilm formation phenotype, both in minimal and rich media. This confirmed its specific contribution to this phenotype and excluded that of the associated secretion defects observed in other secretion systems, which might have also affected biofilm formation. The T1SS mutant showed weaker colonization abilities in a *G. mellonella* infection model compared to the wild-type and complemented strains. Thus, the authors concluded that T1SS contributes to the virulence of *A. nosocomialis*.

The regulation of T1SS in *Acinetobacter* and its secreted effectors is still unstudied. As for the regulation of the identified T1SS effectors, one study showed that the expression of Bap correlated to better biofilm formation, as was expected, but interestingly, it was enhanced by low iron availability^54^. The authors suggested that low iron concentrations might play a role in early biofilm formation and supported their hypothesis in another study that showed how low iron concentrations enhanced the expression of almost 460 genes in *A. baumannii*^55^. Some of these genes were critical to the virulence of *A. baumannii* and were upregulated fourfold under these conditions. Given that the heme acquisition system is a T1SS that is responsible for heme uptake as a source of iron^56^, it would be interesting to know if a homologous system exists in *Acinetobacter* and if an interplay exits between the different types of T1SSs to regulate their functions. High expression of Bap is also detected in an MDR *A. baumannii*^57^ and correlates with higher resistance to desiccation^58^. It would be intriguing to determine if a link between environmental stresses to which *Acinetobacter* is exposed and the expression of T1SS components and substrates could be established in future studies.

## Type V secretion system

The T5SS, also known as the autotransporter system, has the simplest mode of secretion of the systems discussed thus far. Proteins targeted for Type V secretion consist of three major domains, the signal sequence, the passenger domain, and the autotransporter domain^59^. The signal sequence is located at the N-terminal domain of the protein and contains the recognition sequence for the signal peptidase, which is responsible for targeting the protein to the periplasm following Sec pathway secretion^60^. The passenger domain gives the autotransporter its specific effector function. Finally, the autotransporter domain is located at the C-terminal end of the protein, and forms the OM pore through which the protein is secreted to the external environment; thus, the system is seen as spanning only the OM^15,61^. Since the secreted proteins have all the necessary requirements for their own secretion, without the need for accessory elements or an energy generation process, the system is called the autotransporter secretion system.

Many autotransporters have been extensively studied, and many are involved in the virulence of Gram-negative bacterial species. Most notably, the IgA1 proteases of *Neisseria* and *Haemophilus*, which were among the first to be characterized^62,63^, the AIDA-I adhesin of *E. coli*^64^, BrkA of *B. pertussis*, which is involved in adherence, invasion, and serum resistance^65^, and the UspA1 and UspA2 autotransporters of *M*. *catarrhalis*, which are also crucial for serum resistance^66^.

A protein belonging to the trimeric autotransporter (TA) family was first discovered in *A. baumannii* ATCC 17978 using an in silico search and was termed *Acinetobacter* TA, or Ata^67^. It shared many structural features with other TA proteins, such as YadA from *Yersinia enterocolitica* and UspA1 from *M. catarrhalis*, and had the three-domain organization characteristic to T5SS substrates. The C-terminal domain of this Ata protein functioned as the translocator, as it could transport fused heterologous passenger domains to the OM for surface display. Measuring surface Ata using flow cytometry and its transcription using real time reverse transcription PCR (RT-PCR) showed that its production is growth-phase-dependent, with the highest levels observed at early log phase growth. The Δ*ata* mutant formed significantly weaker biofilms than the wild-type strain, suggesting that Ata plays a role in biofilm formation. In addition to its ability to bind extracellular matrix/basal membrane (ECM/BM) collagen types I, II, III, IV, V, and laminin, Ata promoted the adhesion of whole *A. baumannii* cells to collagen type IV, and its lethality in a murine systemic infection model. Forty-four out of seventy-five clinical *A. baumannii* isolates from various types of infections were observed to harbor genes encoding the Ata protein. Almost all of these strains produced Ata, although to varying levels. It would be interesting to investigate if these varying levels of expression also proportionally contribute to the lethality of *A. baumannii*. The same group also evaluated Ata as a vaccine candidate in a subsequent work^68^. Ata antibodies dramatically inhibited the binding of *A. baumannii* to collagen type IV-coated wells and also decreased cell binding to other ECM/BM proteins, such as collagen types I, III, and V and laminin, but the effect was far less pronounced was not significant. The antibodies also significantly promoted opsonophagocytic killing of the wild-type cells in the presence of polymorphonuclear monocytes and human complement. All the tested *A. baumannii* strains were also highly susceptible to the antibody-dependent bactericidal activity of the Ata antibodies in the presence of higher concentrations of human complement (80%). In addition, the antibodies were protective in immunocompetent and neutropenic mice, demonstrating the effectiveness of Ata as a vaccine candidate. Interestingly, in a hypermotile, hyper-virulent *A. baumannii* isolate showing an altered expression profile from the wild-type strain, the autotransporter Ata was overexpressed by 10-fold^26^. This supports previous reports of the role of Ata in adherence, biofilm formation, and virulence.

Although the secretion process of type V autotransporters is quite simple in theory, the process is far from being fully understood. In this effort, Ishikawa et al.^69^ characterized TpgA, a protein encoded by a gene downstream of *ataA* in *Acinetobacter* sp. Tol 5. The authors confirmed that this gene is transcribed in the same operon as *ataA* using RT-PCR. Two domains were identified within the amino acid sequence of the predicted protein, a SmpA domain, which is present in a number of OM lipoproteins, and an OM protein A (OmpA)_C-like domain, which is conserved in a large number of peptidoglycan-associated proteins, such as OmpA. This observation suggested that the protein is a lipoprotein that could interact with both AtaA and peptidoglycans, therefore, it was named TpgA (TAA-associated and PGN-associated protein A). The genetic arrangement of TAAs and TpgA-like proteins is conserved in many Gram-negative species. Interestingly, despite having structural similarities to other OM lipoproteins, the signal peptide of TpgA lacked the characteristic lipoprotein cysteine residue, with the cleavage instead taking place at Ala24, as detected by Edman degradation. This indicated that the protein cannot be a lipoprotein and that it does not interact directly with the OM. However, it possessed all the residues conserved in other peptidoglycan-associated proteins. Unexpectedly, the majority of TpgA localized to the OM fraction, not the periplasm. Since TpgA does not undergo lipid modification, the authors suggested that it could localize to the OM indirectly through AtaA acting as a binding partner. Indeed, TpgA was detected in the periplasmic fraction in Δ*ataA* mutants, and co-localization and pull-down assays with anti-AtaA and anti-TpgA antibodies confirmed this hypothesis. The peptidoglycan-binding activity of TpgA was confirmed because TpgA was detected in peptidoglycan-precipitated pellets of native cell lysates, but not when the peptidoglycan was destroyed by a lysozyme treatment. The adhesiveness of the Δ*tpgA* mutant was significantly lower than that of the wild-type cells, yet it was still higher than the Δ*ataA* mutant. The authors also demonstrated that TpgA is essential for the correct translocation of AtaA to the OM, specifically its passenger domain, and for the surface display of its fibers. The AtaA of *Acinetobacter* sp. Tol 5 was also the subject of structural studies to elucidate the possible reasons behind its non-specific high adhesiveness^70^.

The Ata autotransporter belongs to the T5cSS. Four other T5SSs have been studied: the classical autotransporter pathway (Type Va), the two-partner secretion (TPS) pathway (Type Vb)^71^, a chimera of the Type Va and Vb pathways^72^, and the inverse autotransporter pathway^73^. Recently, more attention has been given to TPS pathways in *Acinetobacter*. These pathways consist of two components transcribed in the same operon, TpsA, which is a large exoprotein, and TpsB, which forms the β-barrel OM structure. Protein A connects at the PORTA domain of protein B for secretion through the OM to either be surface displayed, or to be transported to the extracellular environment^74^. Upon sequencing the genome of *A. baumannii* AbH12O-A2 outbreak strain, characterized by its strong adherence to A549 alveolar epithelial cells, a genomic island carrying the genes for TPS AbFhaB/AbFhaC was observed^75^. The AbFhaB represents TpsA, with a typical Type V signal peptide, an Arg-Gly-Asp triplet (RGD) adhesion motif, and an N terminus hemagglutination activity domain, which is highly conserved in TpsA proteins. The AbFhaC protein belongs to the Omp38/TpsB family, an OM protein with a transport-associated PORTA domain. Wild-type AbH12O-A2 formed weaker biofilms than ATCC 17978 on abiotic surfaces. In contrast, AbH12O-A2 exhibited better attachment and formed biofilm structures on A549 cells, and this phenotype was not observed in the AbH12O-A2Δ*fhaC* mutant. AbfhaB/AbfhaC also mediates the interaction with the host cell fibronectin, although other ECM/BM proteins cannot be ruled out since they were not tested. The system played a role in the lethality of the AbH12O-A2 strain in both an invertebrate and a murine model of infection, thus implicating it in virulence.

## Other secretion systems

The genetic determinants for a contact-dependent inhibition (CDI) system were discovered in *A. nosocomialis* M2^53^. This is a special T5SS in which CdiB is the protein that forms the OM pore through which the CdiA toxin is secreted, with a third component consisting of the protective immunity factor^76^. This system is used to kill neighboring cells lacking the proper immunity protein and is independent from the T6SS. The discovered locus was designated *cdiBAI*^M2^, encoding the A and B proteins and the hypothesized immunity protein CdiI. The authors showed that CdiI is indeed the immunity protein, since the wild-type cells with an intact *cdiBAI*^M2^ locus could kill the Δ*cdiBAI*^M2^ mutant, but not the mutant expressing the CdiI immunity protein in trans. Interestingly, other medically relevant Acinetobacters, such as *A. baumannii* 19606 and 1225, possess two loci encoding for CdiI proteins, named Cdi_1 and Cdi_2, and both are needed for full protection. Two classes of CdiA effectors are generally known for this system, BamA-specific and OmpC-specific, each recognizing a different cell surface receptor^77^. Recently, it was discovered that CdiA uses modular receptor-binding domains as a basis for its surface receptor recognition, leading to the discovery of a third novel receptor, the TsX protein in *E. coli*^78^. The toxic effects of the CdiA protein in *A. baumannii*, and whether they are modulated through the same recognized surface effectors are unknown. However, the need for two, possibly more, CdiI immunity proteins suggests a variety of these toxic effects, and possibly a variety of targets.

T4SSs mediate horizontal gene transfer, and thus play a role in the transfer of antibiotic resistance genes and genome plasticity. A plasmid carrying the genes for the expression of a T4SS was detected in *A. baumannii* strain ACICU^79^. Analysis of the pAB_CC plasmid from *A. baumannii* TYTH-1 led to a similar discovery, and a “*tra*” locus encoding the T4SS components was observed^80^. The authors of this study performed a comparative genomic analysis of the T4SS-encoding loci in a number of clinical isolates involved in hospital outbreaks. Although all the isolates belonged to the European clone II (EC II), their plasmids diverged into two different lineages. The authors hypothesized that these lineages might have emerged even prior to the emergence of the EC II clone. It appears that our knowledge of the T4SS is still in its infancy, and comprehensive work is needed to understand the true role and spectrum of activity of the T4SS in *Acinetobacter*.

## OM vesicles as secretion platforms

OM vesicles (OMVs) are naturally produced by all Gram-negative bacterial species in vivo and in vitro during the different stages of growth^81^. They are typically formed out of LPS, OM proteins and lipids, periplasmic proteins, DNA, and RNA^82^. Based on growth conditions used, OMVs harbor variable cytoplasmic proteins, including toxins, virulence factors, and immune-modulatory proteins, and could be used to deliver these proteins to the host cell^83,84^. Although OMVs are regarded as a pathway for the export of proteins that are not secreted by conventional secretion systems, some secretion system substrates are alternatively secreted by OMVs under certain conditions^85^. The biogenesis of OMVs, as well as the variation in their contents and secretion patterns are the subject of continued research.

In *Acinetobacter*, OMVs were one of the first routes of protein secretion to be studied. A proteomic analysis of OMVs revealed OmpA as one of the major proteins delivered by these vesicles into host cells^82^. A comparison of the composition of OMVs between WT ATCC 19606 and a Δ*ompA* mutant showed that OmpA modulates both the abundance and the composition of OMVs^81^. Following this initial characterization, many groups employed proteomics to identify the proteins secreted through OMVs by various *A. baumannii* strains, especially by comparing the secretion profiles of highly virulent strains versus strains with low virulence^86,87^. These studies mostly reported the variation in the composition of OMVs in terms of abundance and the packaged virulence factors, and the variation usually correlated with the antibiotic resistance and virulence profiles^82^.

In addition to the cytotoxic effects of AbOmpA, OMVs secreted by *A. baumannii* have been shown to illicit a pro-inflammatory response by inducing the expression of cytokines, interleukins, chemokines, macrophage inflammatory protein-1α, and monocyte chemo-attractant protein-1^88^. Since treatment with EDTA and proteinase-K abolished this pro-inflammatory response, the authors concluded that surface-exposed proteins on intact vesicles are responsible for this effect. Deletion of the gene encoding the RNA chaperone Hfq in *A. baumannii* ATCC 17978 resulted in reduced production of OMVs, downregulation of *ompA* gene expression, and an overall reduction in virulence attributes^89^. The modulation of OMVs and their composition by the riboregulator Hfq raises the question of how this relationship is regulated and whether it is directly mediated by Hfq or indirectly through another Hfq effector. OMVs can also mediate the horizontal transfer of entire plasmids harboring multiple β-lactamase-encoding genes that confer antibiotic resistance. The transferred genes include the plasmid-encoded *bla*_OXA-24_, New Delhi metallo-β-lactamase-1 (*bla*_NDM-1_), and extracellular OXA-58, which imparts a protective effect on cabapenemase-susceptible bacteria co-existing with *A. baumannii*^90–92^. Although the mechanism by which the resistance genes are delivered from OMVs is not completely understood, the failure to acquire carbapenemase resistance by a *com*-deficient mutant of *A. baylyi* suggests a role for natural competence^93^.

Since OMVs typically package various membrane proteins that could elicit a strong antigenic response, their use as potential vaccines has been investigated in various species, such as *N. meningitidis*, *V. cholerae*, and ETEC^94,95^. In *A. baumannii*, vaccination with OMVs protected the challenged mice from systemic and lung infections and lowered bacterial loads and circulating levels of pro-inflammatory cytokines^96,97^. OMVs have a natural nanoscale size range and are composed of highly antigenic proteins. These characteristics suggest that OMVs are ideal as a drug-delivery platform, and particularly for vaccine delivery, whose success depends on a robust immunological response. To study this potential, OMVs from the non-pathogenic *E. coli* strain DH5-α were engineered to surface display and deliver a previously investigated antigenic *A. baumannii* protein, Omp22. Immunization with these heterologous OMVs induced a robust and specific antibody response against Opm22 and significantly increased the survival rates of the mice challenged with *A. baumannii*^98^. These results suggest that further optimization of both the OMVs used as the delivery platform and the vaccinating protein could significantly enhance the potential of OMVs as a vaccine delivery system, whether for *A. baumannii* or any other bacterial species.

## Future possible targets to inhibit secretion system functions

The extensive contribution of the different secretion systems to the virulence and physiology of *Acinetobacter* suggests their use as targets for the discovery of new anti-virulence drugs could be a promising future strategy. Except for the HTS for inhibitors of T2SS activity mentioned earlier, no other studies have attempted to utilize secretion systems as drug targets in *Acinetobacter*. However, the idea of targeting secretion systems and their substrates has been studied before, leaving us with a body of experience to guide future work on *Acinetobacter*.

There are many classes of possible secretion system targets that have been previously investigated for drug discovery, including energy generating ATP channels, membrane translocation and assembly factors, and structural elements of delivery systems, such as pili and needles^99^. We will discuss here some of the studies that utilized these targets in other Gram-negative species, providing readers with some insights into how they might be investigated in *Acinetobacter*.

ATPases are interesting targets because, with the exception of T5SS, they are an indispensable component for the secretion process. Another advantage of these targets is that they all show sequence homology and share the same hexameric quaternary structure. The simplicity of measuring ATPase activity makes the assay suitable for optimization of HTS to identify small-molecule inhibitors of the enzymatic activity in compound libraries^100^. ATPase inhibitors prevented the T4SS mediated delivery of the *Helicobacter pylori* major virulence factor CagA and colonization of the gastric mucosa by this bacterium^101^. Taking advantage of the shared homology of secretion system ATPases, it might also be possible to target more than one secretion system with a single molecule. However, the disadvantage of this approach lies in the possibility of targeting other ATPases that are essential for certain physiological functions, transforming these inhibitors into classical antimicrobials. Toxicity in eukaryotic host cells is another expected disadvantage.

A bacterial two-hybrid system based assay was used to screen for small-molecule inhibitors of the interaction between the *V. cholerae* T6SS contractile sheath components VipA and VipB, which are homologous to TssB and TssC in *Acinetobacter*, respectively^102^. These components were chosen as targets owing to their interspecies conservation, in addition to the strictness and specificity of their interaction. Two compounds, KS100 and KS200, were assayed, as were commercially available analogs with low cytotoxicity. The two compounds and some of their analogs considerably inhibited the VipA–VipB interaction at the tested doses, but did not completely abolish it. The compounds markedly inhibited Hcp secretion and were able to completely abolish phospholipase A_1_ secretion, although at a much higher dose than that tested in cytotoxic assays. Using nanobodies to inhibit the interaction between another two key T6SS components, TssM and TssJ, the function of the T6SS was inhibited in vivo^103^.

Accessory components, which play a role in system assembly, represent another attractive target. Examples of accessory molecules successfully targeted for secretion system inhibition are lytic transglycosylases, which despite not being integral structural components of the secretion system machinery, allow the degradation of the PG layer of the cell wall to facilitate membrane assembly^104^. Inhibition of these enzymes could be detrimental to the secretion process, to the extent that targeting the *H. pylori* T4SS LT enzyme rendered the pathogen completely avirulent^105^. PG-modifying molecules have been reported as accessory components for some *Acinetobacter* secretion systems, such as TagX in the T6SS and TpgA in the Ata autotransporter, discussed above in detail. Both proteins were shown to be essential for normal secretion function by both systems, and thus their manipulation as secretion inhibitors could be promising and should be investigated. Molecules required for the proper assembly of secretion systems also include molecular chaperones, which mediate the translocation of folded system components to the OM, such as the GspS pilotins of the T2SS. Although these components have not been identified *Acinetobacter*, it is crucial to investigate how the secretin component of the T2SS is membrane-targeted without these molecules. Given the predicted number of effectors secreted by the T2SS, targeting the membrane assembly process of this system could be a very effective inhibition strategy.

**Fig. 1:**
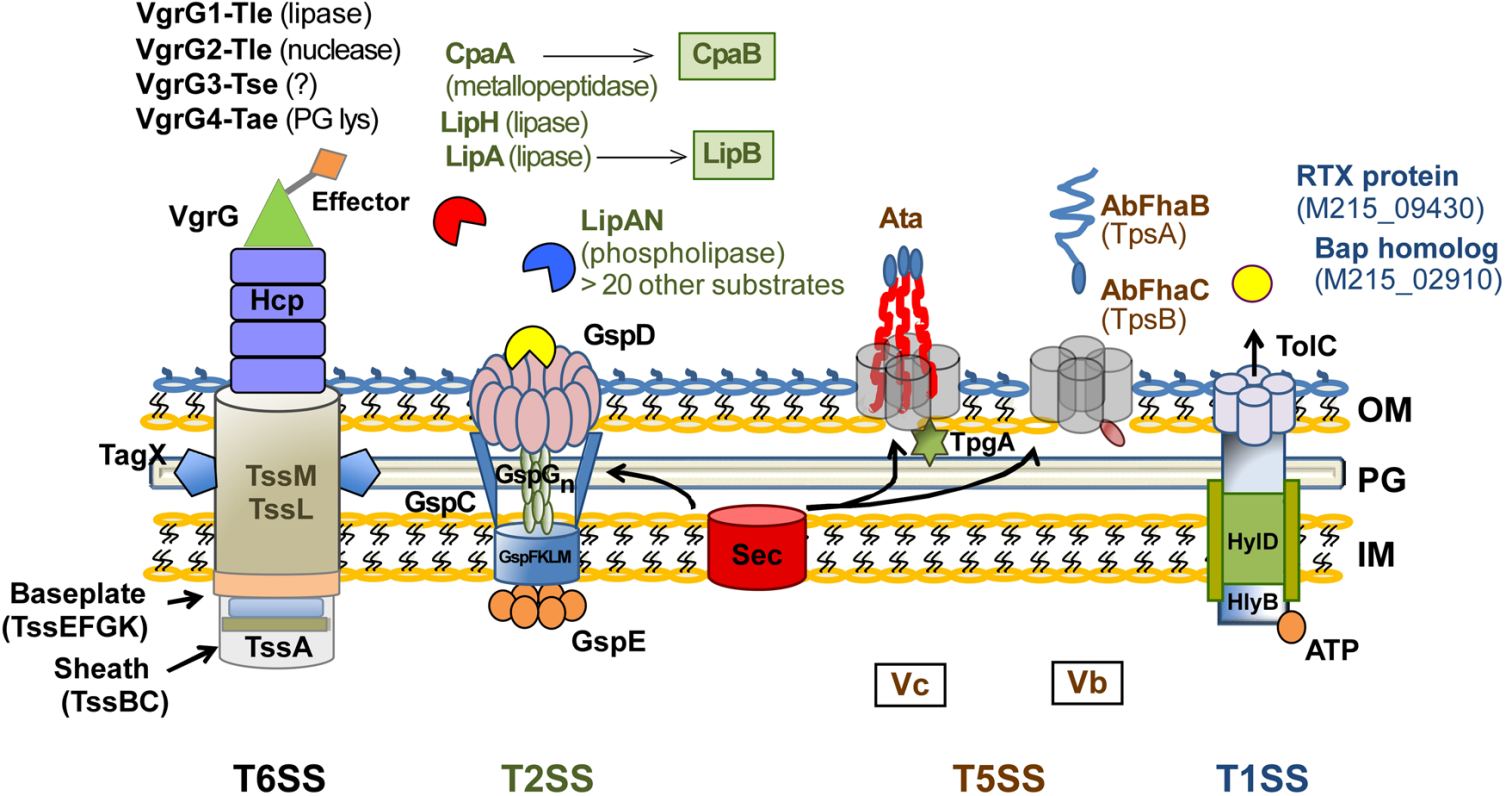
Schematic representation of extensively studied secretion systems in *Acinetobacter* species, showing their general architecture, membrane components, and the most important effectors. T6SS effectors are *vgrG*-associated and are encoded by genes that are in close proximity to each of the four *vgrG* genes identified in *A. baumannii*. The VgrG1-associated, VgrG2-associated, and VgrG4-associated effectors are type VI lipase effector (Tle), a type VI DNAse effector (Tde), and a peptidoglycan-targeting type VI amidase effector (Tae), respectively. The VgrG3 type VI effector (Tse) does not possess characteristic functional domains and is predicted to have a role in bacterial killing. A large number of T2SS effectors have been identified, including the verified lipolytic enzymes LipAN, LipH, and the chaperone-dependent LipA (chaperone LipB), in addition to chaperone-dependent CpaA metallopeptidase (chaperone CpaB). More than 20 other predicted T2SS effectors are yet to be verified as true substrates. Two T5SS subtypes have been identified in *A. baumannii*, the *Acinetobacter* trimeric autotransporter (Ata) and the two-partner secretion (TPS) system AbFhaB/FhaC, which plays a role in cellular adhesion. Both the T2SS and T5SS depend on the Sec machinery for the translocation of substrates across the inner cytoplasmic membrane (IM). In addition, *A. baumannii* possesses a homolog of the TolC-HlyB-D T1SS, which secretes a hemolysin-like RTX protein and a Bap-like protein important for biofilm formation

Sec secretion is the preliminary step for both T2SS-mediated and T5SS-mediated secretion (Fig. 1). Targeting Sec secretion could be tricky, as it also mediates the secretion of molecules that are essential for a number of physiological targets. Thus, targeting Sec secretion could create a selective pressure for resistance. Moir et al.^106^ developed a whole-cell bioluminescent reporter assay to screen for inhibitors of T2SS-mediated inhibition. The downregulation of a lac-regulated *secA* gene on the complementation plasmid in a *secA* mutant of *P. aeruginosa* or *B. pseudomallei* was used to identify the genes showing elevated expression under these conditions. The promoters of these genes were fused to the *luxCDABE* operon to respond to depletion of SecA by increased bioluminescence. These reporter strains were then used to screen for SecA/T2-mediated inhibition of secretion. Identified compounds did not inhibit Sec-mediated β-lactamase secretion into the periplasm, but instead inhibited extracellular T2SS-mediated secretion of elastase and phospholipase C. Although the assay requires a secondary assay to confirm the specificity of the SecA/T2 secretion inhibition, it could represent a valuable tool to screen for T2SS inhibitors.

While redundancy of secretion system components and the substitution of secretion pathways might compromise the strategy of targeting these systems for drug delivery, targeting the most important effectors is a logical alternative. One of the earliest of these studies is a HTS to identify inhibitors of cholera toxin secretion^107^. A small-molecule named virstatin was identified that inhibits the ToxT transcription factor, which controls the expression of the Ctx-encoding gene. Interestingly, a virstatin-resistant *V. cholerae* variant was isolated during the screen, showing the impressive genomic plasticity of these pathogens in response to the pressure of antimicrobials, even targeting non-essential physiological components.

## Concluding remarks

Over the past decade, efforts from several research groups have unraveled the role of different secretion systems in *Acinetobacter*. In particular, a considerable body of knowledge has accumulated for T2SS and T6SSs. It should be noted that during the writing of this manuscript, a review by Weber et al.^108^ on the subject of secretion platforms in *Acinetobacter* has been published, as well as a manuscript providing insights into our current knowledge of the T2SS in *Acinetobacter*^37^. In this review, we extensively discuss novel findings for all secretion systems identified in *Acinetobacter* species, along with their subtypes and possible regulatory elements. We also highlight their application as potential drug targets and discuss possible targeting strategies and obstacles.

The structural components of the different types of secretion systems are highly conserved across the different species of *Acinetobacter*. However, there is not enough data on the differential roles they play and the nature of the secreted proteins across these species. It is crucial to compare the secretomes of secretion systems, in particular those of types I, II, and V, between highly virulent and less virulent strains, as well as across strains with contrasting antibiotic resistance profiles. These assays would provide us with a better understanding of the evolution of *A. baumannii* into such an aggressive pathogen, and would be useful for the process of drug development.

Given the evidence from the body of research using the different infection models, we believe that the T6SS does not contribute to the virulence of *A. baumannii*. However, characterizing additional effectors and identifying the still unknown functions of some of the currently known ones could elucidate the full scope of its functional role. All other secretion systems showed varying degrees of contributions to the virulence of *Acinetobacter*.

A poorly tackled area of research is the contribution of secretion systems to the antibiotic resistance of *A. baumannii*. In this regard, OMVs and the T4SS could be major contributors to the transfer of resistance-encoding elements or even fully functional enzymes. However, these studies should be tightly controlled, since the perturbation of secretion systems usually disrupts membrane integrity, which is likely to influence the MIC of antibiotics. Another area of particular interest is the discovery of homologs for other secretion systems, such as subtypes of type I and V secretion systems, given the crucial roles they play in the physiology and virulence of other bacterial species.

Thus far, little is known concerning the regulation of secretion systems in *Acinetobacter*, especially with respect to pathogenesis. The best-studied example is the regulation of the T6SS through the gain and loss of plasmid DNA carrying the T6SS regulatory elements and its relation to fitness and antibiotic resistance. Another observation is the common downregulation of secretion system components in cases of events resulting in membrane disruption, probably to lessen the pressure on this vital cell component and maintain as much membrane integrity as possible. A very interesting topic of study is the relationship between the T2SS and serum sensitivity and whether this is directly regulated through the components of the system or through one or more of the secreted proteins.

The study of secretion systems in *Acinetobacter* has provided us with insights into the success of this fascinating bacterial genus as a human pathogen. In the future, the discovery of new secreted effectors and an understanding of how they are regulated will provide a clearer picture for their contribution to the pathogenicity of *Acinetobacter* species.
